# Mechanical Behaviour of As-Manufactured and Repaired Aligned Discontinuous Basalt Fibre-Reinforced Vitrimer Composites

**DOI:** 10.3390/polym16081089

**Published:** 2024-04-13

**Authors:** Leon L. Messmer, Ali Kandemir, Burak Ogun Yavuz, Marco L. Longana, Ian Hamerton

**Affiliations:** Bristol Composites Institute, School of Civil, Aerospace, and Design Engineering, University of Bristol, Queen’s Building, University Walk, Bristol BS8 1TR, UK; kf19907@alumni.bristol.ac.uk (L.L.M.); ogun.yavuz@bristol.ac.uk (B.O.Y.); marcoluigi.longana@polimi.it (M.L.L.); ian.hamerton@bristol.ac.uk (I.H.)

**Keywords:** short fibre reinforced composites, HiPerDiF, micro-mechanical characterisation, interfacial shear strength

## Abstract

The aim of this research is to investigate basalt as a natural mineral-based fibre together with a vitrimeric resin as a sustainable alternative to standard composite materials. Vitrimers combine the properties of thermoset and thermoplastic polymers, enabling the repair of specimens and hence prolonging the lifetime of the composite material. The micro-mechanical characteristics between the basalt fibres and the vitrimer resin are reported and shown to match those of a standard Skyflex K51 epoxy resin. Discontinuous (4 mm) basalt fibres were employed to produce aligned discontinuous fibre-reinforced composites (ADFRCs) using the high-performance discontinuous fibre (HiPerDiF) technology. The mechanical characteristics of the laminates were investigated through tensile testing and the fracture zones were analysed under a scanning electron microscope. By normalising the results by their respective fibre volume fraction, it was discovered that the vitrimer–basalt ADFRCs exhibited, on average, a 4% higher strength and a 25% higher stiffness compared to their basalt epoxy counterparts. The repair potential of the vitrimer ADFRC specimens was explored during low-temperature compression repair. Two approaches were tested using double-sided local- and full-patch repair. Both successfully recovered a significant amount of their prime strength. In conclusion, the potential of the sustainable vitrimer–basalt composite is shown by its competitive mechanical performance. Combining this with the manufacturing flexibility, repair potential, and recyclability of the material, the vitrimer–basalt composite seems to be a competitive alternative to standard glass epoxies.

## 1. Introduction

During the last three decades, the composite industry has witnessed drastic growth, which is primarily attributed to the high specific strength and tailoring capabilities inherent to fibre-reinforced polymer (FRP) composites [1]. The global composite market size was evaluated to be an estimated USD 93.69 billion in 2022, with a compound annual growth rate of 7.2% [2]. The FRP applications cover a wide spectrum of industries including aerospace, transport, marine, renewable energies, sports, and the automotive sector to name a but few [1,3,4,5,6].

In the European composite industry, glass fibres are by far the most commonly used reinforcement, and in 2018 were incorporated into over 95% of the total volume of composites produced [7]. In the automotive and marine industry, glass fibres are commonly coupled with epoxy matrices to form “synthetic” composites (i.e., wherein both the fibre reinforcement and polymer matrix are derived from mineral deposits or petroleum distillates) [4,8,9]. These petroleum-based non-renewable materials are complex to recycle/down-cycle and prevent composites from being truly sustainable [10].

As sustainability is becoming more urgent due to anthropogenic climate change, producers and consumers have been transitioning to so-called “greener” materials [9]. Coincidentally, basalt, a mineral-based, natural material, is frequently referred to as a more sustainable alternative to glass [11]. The term basalt summarises a group of stones and minerals that are of volcanic origin. These rocks are crushed, molten, and drawn into fibres [12]. Basalt fibres offer good modulus, improved strain to failure, and equal to higher strength than glass analogues [13]. Furthermore, the fibres prove to have outstanding environmental resistance such as high-temperature tolerance, good acid and alkali resistance, and are non-toxic and easily processed [14,15]. Basalt is an abundant material, which leads to a cheap fibre price. The price of basalt fibres is comparable to that of glass fibres [14].

As an alternative resin system, in the past decades, reversible bonds were introduced into polymeric networks, hence making these thermosets reprocessable, healable, and recyclable [16]. It was discovered that polymer networks with reversible covalent bonds can yield substantial mechanical properties. This group of materials are also known as covalent adaptable networks (CANs) [17]. However, it was a challenge to have the CANs reform the covalent bonds while they were dissolving at the same time. In 2011, Leibler et al. published his work on CANs that maintained their crosslink density at high temperatures and hence remained stable [17]. This allows the material to be reshaped in a similar fashion to a thermoplastic material while new covalent bonds keep regenerating, resulting in a quasi-constant crosslink density. This material group was named vitrimers and is also referred to as associative CANs [18,19]. Vitrimers combine the characteristics of thermoset and thermoplastic materials to yield strong, durable, and easily processable properties such as a thermoset. Yet additionally, they have end-of-life reprocessability similar to a thermoplastic [20,21].

When it comes to recycling composites, one of the major challenges is to separate the matrix from the fibres. Mallinda Inc. developed a vitrimeric resin that can be fully separated from the fibres using reagents that are utilised in the initial resin synthesis [16,22]. This allows for the recovery of the fibres and resin separately without significantly deteriorating the properties of either component. Another sustainable solution is reclaiming the fibres by using pyrolysis or solvolysis treatment [23,24].

One crucial step of fibre recycling is the repurposing of reclaimed fibres [10]. One way to confront this challenge is to realign the fibres; this can be complex due to fluffing and entanglement of the fibres. One sustainable approach to realigning them is the High-Performance Discontinuous Fibre (HiPerDiF) method, which was invented at the University of Bristol and subsequently patented [25]. This method uses discontinuous fibres and manufactures them into aligned discontinuous fibre (ADF) preforms. ADFs prove to have similar mechanical properties compared to their continuous counterparts in the features of high alignment and optimum critical fibre length [26]. The HiPerDiF method operates with water as an alignment medium, which further reduces the environmental impact [27]. It was proven that through the HiPerDiF approach, the ADFRP’s tensile modulus, strength, and failure strain were close to that of the equivalent continuous fibres [25]. The method was demonstrated with synthetic fibres, such as carbon, as well as with natural fibres including flax [27,28]. ADFs can be implemented to provide superior formability, which reduces manufacturing waste [29].

Recent research on vitrimers categorised the properties of the resin combined with various fibres including carbon, glass, and natural fibres [16,20]. This research project aimed to expand on this field of research by categorising the micro-mechanical characteristics between basalt fibres and vitrimeric resin and comparing them to a standard epoxy resin. This was performed by single-fibre tensile testing, microbond testing, and determination of the critical fibre length. Following which, basalt fibres were introduced into the HiPerDiF process for the first time. The aim was to prove that the material is suitable for the HiPerDiF process and how two different sizing types would affect the manufacturing approach. Sizing is a thin surface coating which is applied to fibres during the manufacturing process that protects the fibres, improves adhesion with matrices, and enhances environmental resistance [30]. In an endeavour to highlight a potentially sustainable alternative to the typical composite material, which could propose a potential circular economy, the HiPerDiF ADFs were combined with a vitrimeric resin, and an epoxy resin, separately, as a comparison. The fracture mechanics of the different material couples were investigated under a scanning electron microscope (SEM) to further widen the understanding of ADF composites. To unveil the repair potential of this composite system, two novel repair approaches were being explored in this research: double-sided local- and full-patch repair. The resin flow during the repair process was examined and the fibre amount in the fracture zone was analysed, aiming to further understand the repair process and enhance the repair potential of ADF vitrimer composites.

The objective of this research paper is to categorise the micro-mechanical characteristics between the two types of basalt fibres and the vitrimer matrix, and to compare it to an epoxy equivalent, to validate basalt as a suitable material for the HiPerDiF process, prove vitrimeric resin to be a suitable material to manufacture ADFRCs, and to investigate two repair approaches for the vitrimer ADFRCs. Furthermore, it is aimed to make a step toward the circularity of composites by providing additional insights into fibre reusability, a novel fibre–matrix system, and enhancing the life cycle of ADFRCs by underlining their repair potential.

## 2. Micro-Mechanical Characterisation of Basalt Composites

### 2.1. Materials and Methods

Basalt fibres with two different sizing types from the Deutsche Basalt Faser GmbH (Sangerhausen, Germany) were investigated. The first sizing type was the A76.9.2, which is based on silane with a glycidoxypropyl functional group and hydrolysable methoxysilyl groups. The second sizing type is the A76.9.4 sizing, which is based on silane with a reactive primary amino group and hydrolysable ethoxysilyl groups. They will be referred to as sizing type 1 and sizing type 2, respectively. Continuous tows and 4 mm long discontinuous fibres having an approximate diameter of 20 μm were examined. These were used in the HiPerDiF machine to produce fibre preforms.

Skyflex K51 Epoxy resin (SKChemicals, Seoul, Republic of Korea) was selected as a typical thermoset epoxy resin. Resin strips were chosen over a liquid form due to their good handleability, higher precision in fibre volume fraction tailoring, and diversity during manufacturing. 150 mm × 5 mm strips were cut to size using a ply cutter to ensure consistent resin quantity across the specimens.

For the microbond testing, Prime 27 epoxy resin (Gurit, Zurich, Switzerland) was chosen as a standard epoxy due to its easy handability and easier application on the fibres.

VITRIMAX T100, a polyimine-based vitrimer produced by Mallinda Inc. (Denver, CO, USA), was chosen to be moulded, reshaped, and repaired. It combines the characteristics of thermosets and thermoplastics while maintaining good mechanical properties [31]. The resin blend was prepared as a (2.5:1) hardener–resin mixture and cured as a thin film in the oven at 135 °C for 60 min. Once cured, the film was heated up and cut into adequate resin strips of dimensions 150 mm × 5 mm for further manufacturing.

To determine the suitable fibre length for the HiPerDiF process, the critical fibre length of the resin fibre couples was required.

The critical fibre length is a concept taken from the yielding behaviour of metals and applied to discontinuous fibre-reinforced plastics [32]. It describes the minimal fibre length required to ensure fibre breakage-dominated failure [33]. If the length of the fibres is below the critical length, fibre pull-out may occur, which results in lower performance. For ADF composites to yield comparable properties to a continuous counterpart, the fibre length must be significantly larger than the critical fibre length. To determine what fibre length was required for the HiPerDiF process, the critical fibre length was calculated using the following formula:

(1) $$l_{c}=\frac{d\cdot \sigma_{f}}{2\cdot \tau }$$

where *d*, $\sigma_{f}$, and τ represent the fibre diameter, the fibre strength, and interfacial shear strength (IFSS), respectively. To obtain the fibre strength, single-fibre tensile testing (SFTT) was performed. For the interfacial shear properties, microbond testing was carried out.

For the single-fibre tensile testing, specimens of the two fibre types were prepared by placing individual continuous fibres between two plastic end tabs and securing them using super glue. The specimens had a gauge length of 12 mm to comply with the ASTM standard C1557-20 [34]. This also minimises fibre misalignment issues compared to smaller gauge lengths [35]. A Dino-Lite AM3113T optical microscope (AnMo Electronics Corporation, New Taipei City, Taiwan) was used to record the diameter at three different points along the individual fibres. These values were averaged for each fibre to obtain the fibre strength as follows:

(2) $$\sigma_{f}=\frac{F}{\pi \cdot {\left(\frac{d}{2}\right)}^{2}}$$

where *F* and *d* represent the failure load and the fibre diameter, respectively.

A Dia-stron LEX820 Extensometer (Clarksburg, WV, USA) equipped with a 20 N load cell was used to apply tension to the fibres until failure. The test was performed at quasi-static speed with a displacement speed of 2 mm min^−1^ as outlined by the ASTM standard. For each fibre type, 20 specimens were prepared and tested.

**Figure 1:**
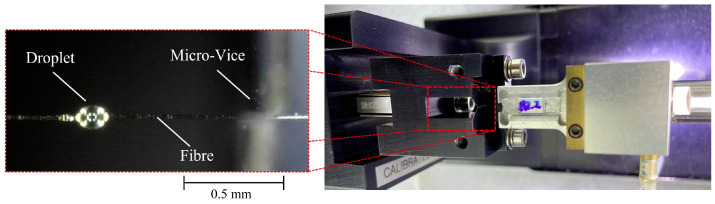
Microbond testing setup-extensometer equipped with a 30 μm micro-vice.

To determine the interfacial shear properties between the basalt fibres and the two different matrice types, microbond testing was performed on the various fibre–matrix couples. The microbond testing was performed according to the method established by Gaur et al. [36]. The test setup is shown in Figure 1.

Individual continuous fibres were mounted between plastic end tabs using super glue. Once dried, for the epoxy resin, single droplets of Prime 27 epoxy resin were applied using a single glass fibre. The matrix was mixed using the extra slow hardener in a mass ratio of 100:28 according to the material data sheet. The droplets were cured in an oven at 80 °C for 12 h.

For the vitrimer resin, the droplets were applied in a similar manner. The resin blend was prepared as a (2.5:1) hardener–resin mixture and the droplets were cured on the fibres in the oven at 135 °C for 60 min. It was aimed to vary the size of the droplets to capture a larger spectrum of droplets.

The droplet size, position, and fibre diameter were recorded using a Dino-Lite AM3113T optical microscope. The specimens were mounted in a Dia-stron LEX820 Extensometer. The extensometer was equipped with a micro-vice to capture the droplet while tension was applied to the fibre. This allowed the application of pure shear onto the droplet and debonded it from the fibre. The test was performed at a quasi-static speed of 2 mm min^−1^ to comply with the standard set by Gaur et al. [36].

Initially, a 50 μm micro-vice was used; however, after observing repeated droplet fracture, the micro-vice was switched to 30 μm. This captured the droplet better and led to successful debonding of the droplet. The Dino-Lite microscope enabled observation of the test and the categorisation of the result. The successful debonding of the droplet enabled the IFSS to be obtained using the following formula:

(3) $$\tau =\frac{F}{\pi \cdot l_{d}\cdot d_{f}}$$

where *F*, $l_{d}$, and $d_{f}$ denote the debonding force, the droplet length, and the fibre diameter.

For each fibre–matrix couple, more than 90 droplets were prepared. Successful droplet debonding was observed in 28 and 25 cases for the sizing types 1 and 2 with the epoxy resin, respectively. For the vitrimer successful droplet debonding was observed in 13 cases for each of the two sizing types. The tested droplet width ranged between 53 and 169 μm with an embedded length varying between 72 and 241 μm.

### 2.2. Results and Discussion

Table 1 summarises the results of the SFTT. Some fibres broke before the test due to the loading procedure and the fragile nature of individual fibres. The fibres which were successfully tested fractured in the gauge length and yielded a linear stress–strain response as expected. It can be noted that the properties are close to equivalent between the two fibre types. This is due to the fibres being made from the same basalt and only the sizing agent being different. Sizing agents affect the interfacial shear properties more than the mechanical properties of the fibres.

As a comparison, the SFTT results of HYBON 2026 glass fibres, reported by Messquita et al., were added [35]. These were investigated using a similar approach. In contrast to the literature findings, it seems that the E-glass fibre has a higher tensile strength and elastic modulus than the basalt equivalent.

During the microbond testing, three responses were observed. Either the fibre failed before the droplet could debond (fibre failure), the matrix cracked and the droplet broke (matrix failure), or the droplet successfully debonded from the fibre. This response enabled the calculation of the IFSS between the matrix and the fibre.

The results of the microbond test were analysed (see Figure S2). The successful debonding results were plotted and using a linear fit having an intercept at 0, the IFSS ($\tau_{fit}$) was determined. This was compared with the average IFSS of the droplets $\tau_{AvG}$, which was calculated using Equation (3). Additionally, the 95% confidence interval was plotted. The comparison between $\tau_{fit}$ and $\tau_{AvG}$ is reported in Table 2.

It was found that the average and the fitted value for the IFSS show consistency between each other. During the testing, it was observed that the matrix failure was most likely to occur when the micro-vice was significantly bigger than the width of the droplet. This led to a load transfer on the edge of the droplet, which ultimately led to the cracking of the droplet itself. This was mostly observed for smaller droplets (width < 65 μm). When the micro-vice was smaller, the droplets were gripped at their root, which led to better shear load transfer, which catalysed successful debonding. It is important to note that the micro-vice should not be too small either; otherwise, the fibre can be damaged or surrounding smaller droplets might interfere with the test. Fibre failure was most frequently observed when the embedded area was above 0.01 mm^2^. In these cases, the debonding load approached the fibre strength, which led to fibre failure.

Table 2 summarises the resulting IFSSs and critical fibre lengths of the respective fibre–matrix couples. It was noted that the basalt fibres with the second sizing type seemed to have significantly better adhesive properties with the vitrimer than the alternative sizing type. When looking at the IFSS with epoxy, both sizing types perform close to equally. When comparing the vitrimer and the epoxy, the results show that the vitrimer–basalt couple has an 8% and 37% higher IFSS than the epoxy equivalent for the sizing type 1 and 2, respectively. This led to a smaller critical fibre length for the vitrimer–basalt couples.

To conform to the principle that the discontinuous fibres are required to be significantly larger than the critical fibre length, an estimate of ten times larger was applied to the smallest critical length determined, and it was rounded to the next millimetre [37]. It was determined that 4 mm was a suitable fibre length for the discontinuous fibres, which was also a suitable fibre length for the dispersion in the alignment medium of the HiPerDiF process.

## 3. Composite Manufacturing and Testing

### 3.1. Materials and Methods

To manufacture the composite specimens, the 4 mm basalt fibres were run through the water-based HiPerDiF machine to produce aligned discontinuous fibre (ADFs) preforms [37]. To determine the aerial weight of the preforms and the materials, three specimens of each material type were weighed and their area was determined using a high-resolution Epson Expression 11000XL scanner (Hemel Hempstead, UK). The areal weights (expressed in grammes per square metre, GSM) data are reported in Table 3. Based on the layup sequence, the values were used to predict the fibre volume fraction of the later manufactured specimens; this is further elaborated in the Supplementary Information.

Two epoxy resin strips and one aligned 4 mm discontinuous basalt fibre preform were combined to form a pseudo-prepreg in a consolidation machine. This made the aligned discontinuous fibres easier to manipulate. Six layers of the pseudo prepreg were laid up in a semi-closed mould having dimensions 150 mm × 5 mm. The mould was then vacuum-bagged and the material was cured in an autoclave. The first dwell occurred at 85 °C for 85 min and a second dwell began at 127 °C for 60 min while a pressure of 6 bar was applied according to the Skyflex K51 epoxy data sheet. Thermal couples were implemented to ensure that the cure cycle the material experienced corresponded to that outlined by the datasheet. Next, 5 cm glass fibre end tabs were applied using Dymax 3139 adhesive (Torrington, CT, USA) to mitigate residual stresses due to the tensile testing machine’s compression and the specimens’ thin nature ranging between 0.6 and 1 mm.

For the vitrimer–basalt composite, the vitrimer film was cut into 150 mm × 5 mm strips whilst heated at 60 °C. For each specimen, five basalt ADF preforms were sandwiched between six resin strips in the semi-closed mould. The layup was performed at a temperature of 60 °C to allow better formability of the resin strips. The specimens were consolidated in the oven under vacuum for 4 h at 140 °C. This allowed for the resin to thoroughly wet the fibres, and to consolidate accordingly. It is important to note that the oven was chosen over the autoclave due to the reduced energy usage for a more sustainable process. No end tabs were required due to the thicker nature of the vitrimer–basalt specimens ranging between 1.7 and 2.0 mm.

To comply with the ASTM D3039/D3039M-17 standard, at least 5 specimens of each resin fibre couple were tested [38]. The tests were performed in a Shimadzu (Columbia, SC, USA) tensile test machine at a quasi-static speed of 1 mm min^−1^. The electromechanical test machine was equipped with a 10 kN load cell and the displacement was recorded using a video extensometer. The testing machine gripped the specimen at 40 mm on either side, leaving a gauge length of 70 mm.

To determine the fibre volume fraction of the specimens, the thermo-gravimetric analysis (TGA) approach established by Yee et al. was implemented [39]. The analysis was performed using a simultaneous thermo-gravimetric analyser (STA 449 F3 Jupiter, NETZSCH, Selb, Germany). Five 20 mg samples were taken from various specimens for each fibre–matrix couple. Each sample was placed in its own crucible. The crucible was heated to 250 °C at a rate of 20 °C min^−1^ followed by heating 10 °C min^−1^ to 600 °C. The specimen was kept at 600 °C for 40 min. The process occurred in an inert gas: in this case, nitrogen. This allowed the resin to vaporise and the fibres remained in the crucible. Based on the matrix and fibre density, the fibre volume fraction was then determined. This method assumes that the voidage in the composite specimens was negligible. The results of the TGA are given in the Supplementary Information in Table S2.

To observe the fibre alignment of the locally repaired specimens, 20 mm sections of the repaired zone and virgin cross-sections were potted in resin, grinded, and polished according to the standard approach of micro-structural preparation [40].

### 3.2. Results and Discussion

**Figure 2:**
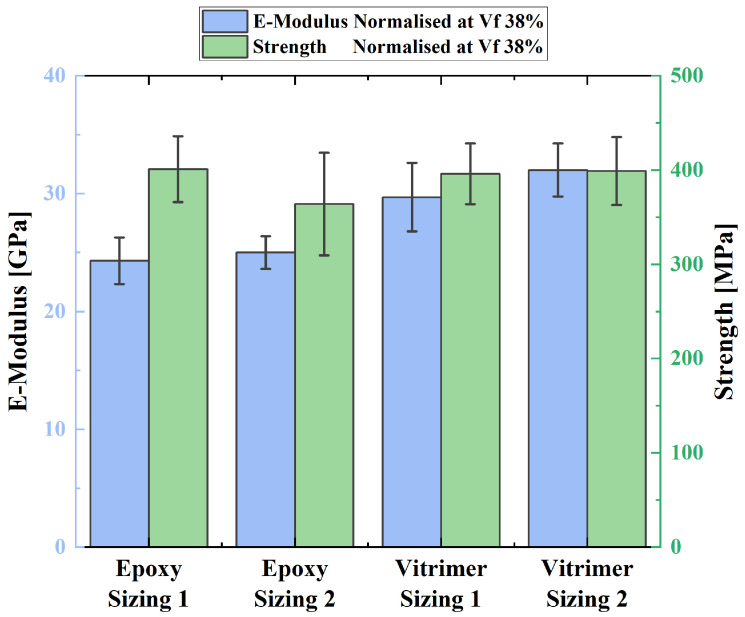
Graph representing the normalised tensile test results at a Vf of 38%. Error bars represent the standard deviation from the mean.

All tensile tests were successful, and the specimens fractured in the gauge length. All results were normalised to a common fibre volume fraction of 38% using a TGA to allow to make a meaningful comparison. A fibre volume fraction of 38% was chosen as this is a reasonable fibre volume fraction achievable with the HiPerDiF as stated in previous work of the HiPerDiF team [25]. The results are shown in Figure 2. Additionally, all resulting stress–strain curves (see Figures S3 and S4) and the results from the TGA are presented (see Table S2) in the Supplementary Information.

Figure 2 shows that the vitrimer–basalt specimens appear to match the performance of the epoxy basalt. The tensile strength of the two resins combined with the sizing type 1 basalt seems to be equal. The average tensile strength of the vitrimer specimens lies at around 396 and 399 MPa for the type 1 and type 2 sizing, respectively. The second sizing type basalt seems to perform moderately better with the vitrimer resin compared to the epoxy resin. The vitrimer and the second sizing type fibres couple is around 35 MPa stronger than the equivalent epoxy composite. The vitrimer fibre couples appear to be stiffer than the equivalent epoxy fibre couples. The elastic modulus of the vitrimer samples is around 6 GPa higher than that of the epoxy equivalent.

It was found and proven that basalt fibres are a suitable material for the HiPerDiF procedure. The critical fibre length was determined and tested in the HiPerDiF machine. It was shown that basalt preforms of comparable quality to the previously tested materials are achievable [37].

While comparing the normalised tensile test results, it is important to acknowledge the limitation between the preform quality. The aerial weight of the preforms may be similar, but the alignment quality of the ADF may differ. Additionally, scaling the mechanical properties of specimens with significantly different fibre volume fractions to a common one has its limitations. Under the scanning electron microscope (SEM) (Hitachi TM3030Plus tabletop Microscope, Hitachi High-Tech, Tokyo, Japan), the high fibre volume fraction epoxy specimen’s failure mode was observed to be fibre-dominated. The vitrimer specimens on the other side showed more of a hybrid failure mode. A combination of fibre failure and fibre pull-out was observed (the latter being an interface failure mode). Nonetheless, it is reasonable to conclude that basalt fibres yield better or comparable mechanical properties combined with the vitrimer than with the epoxy, as a hybrid failure mode would result in reduced performance. The better performance of the vitrimer and the basalt was also underlined by the superior IFSS. This emphasises the potential of vitrimer as a sustainable alternative to standard epoxy resin.

## 4. Vitrimer Composite Repair Work

**Figure 3:**
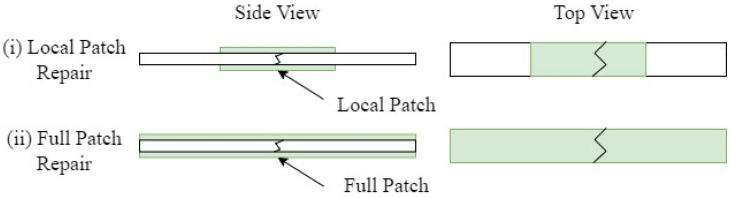
Schematic depicting the two repair approaches: (**i**) Local-patch repair approach, (**ii**) Full-patch repair approach.

**Figure 4:**
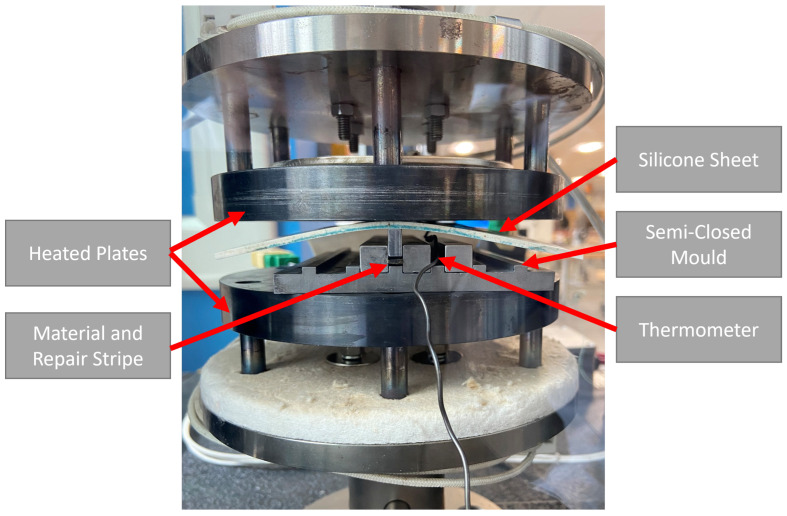
Specimen repair setup in the Instron machine equipped with the two heated plates and the semi-closed mould.

To repair the broken vitrimer specimens, two different approaches were investigated. Approach (i) uses two 40 mm × 5 mm local repair strips and (ii) uses two 150 mm × 5 mm full-length repair strips; both approaches are schematically depicted in Figure 3. The repair patches consisted of one preform layer and two vitrimer resin layers and were manufactured according to Section 3.1. Both repair procedures were performed in an Instron electromechanical testing system (Norwood, NC, USA) equipped with a 50 kN load cell. The semi-closed mould, containing one specimen and the respective repair patches, was placed between two heater plates as seen in Figure 4. A silicon sheet was used for homogeneous pressure application onto the specimen. The temperature of the mould was elevated to 120 °C; once the desired temperature was reached, 0.69 MPa of pressure was applied for 5 min.

### 4.1. Vitrimer Specimens Repair Methodology

Owing to the presence of the local repair patches in approach (i), the consolidation pressure was applied unevenly over the specimen. After 5 min it was observed that the specimen had curved due to the non-homogeneous consolidation pressure. The repair time was therefore increased to 20 min for approach (i) to ensure recovery of the initial geometry and good consolidation of the broken section. The additional repair time was determined by visual inspection of the specimen, and iterating through a variety of repair times. The repaired specimens were tested as previously stated in Section 3.1.

### 4.2. Results and Discussion

#### 4.2.1. Repair of the Vitrimer Specimens

**Figure 5:**
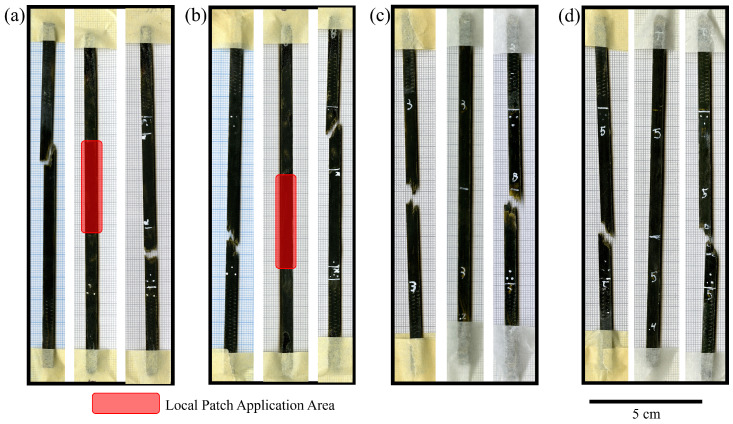
Scans of the virgin specimens, the repaired specimens, and the repaired specimens after tensile testing in that respective order from left to right: (**a**) Sizing type 1 local-patch repair. (**b**) Sizing type 2 local-patch repair. (**c**) Sizing type 1 full-patch repair. (**d**) Sizing type 2 full-patch repair.

All specimens were successfully repaired. Minimal dry spots were detected and only minimal leakage of resin was observed, as shown in Figure 5. Increases in thickness of the specimens due to the addition of the repair patches were observed. The repaired specimens of the full-patch repair were on average 49% and 46% thicker than their virgin counterparts for the sizing type 1 and 2, respectively. For the local-patch repair, a more moderate average thickness increase of 19% and 16% was observed for the sizing type 1 and 2, respectively.

It is noted that the projected fibre volume fraction of the repair strips was lower than that of the virgin material. Nonetheless, the fibre volume fractions of the virgin and repaired specimens are comparable when looking at the standard deviation as shown in the Supplementary Information. The reason of the maintenance of fibre volume fraction could be a slight amount of resin bleeding out of the mould. The fibre volume fraction through the cross-section might vary due to the absence of mixing between the virgin and repair material.

**Figure 6:**
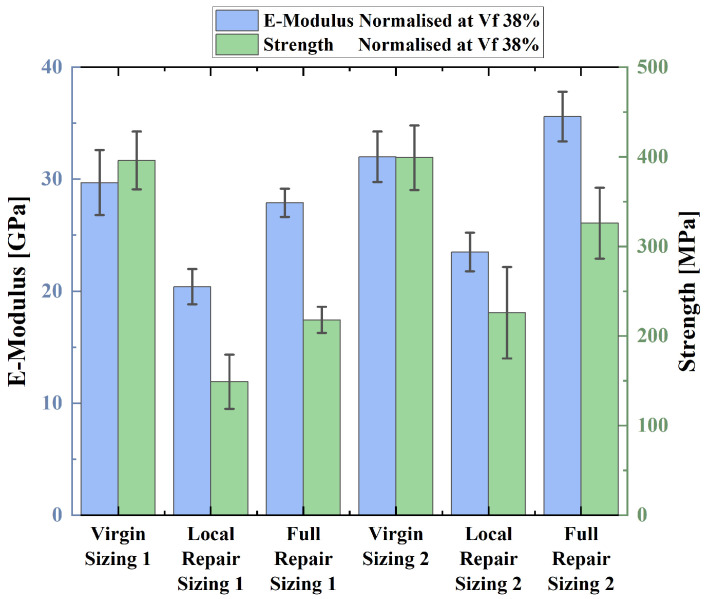
Normalised tensile test results of the repaired vitrimer specimens at a common fibre volume fraction of 38%. The error bars indicate the standard deviation from the mean.

The tensile test results of the repaired specimens were normalised to a common fibre volume fraction of 38%. The results are shown in Figure 6. The full-patch repair approach recovered 57.1% and 78.2% of its initial strength for the type 1 and type 2 sizing, respectively, as reported in Table 4. This is a better recovery than the local-patch repair approach, which recovered 34.0% and 56.1% for the type 1 and type 2 sizing. Additionally, the full-patch repair matched the initial stiffness of the virgin specimens. In contrast, the stiffness of the locally repaired specimens appeared to be reduced by around 29%.

When comparing this to the previous work, investigating single-lap joint repair of aligned discontinuous flax fibre vitrimer composites, a slight improvement in the recovered properties was observed. A strength recovery of 67% was recorded and the specimens broke in the same area as previously [28]. The double-sided local-patch repair approach explored here showed failure in different regions, indicating recovery of the initial fracture zone to a similar level to the surrounding material.

In contrast, Niedrnhuber et al. investigated thermoset composite repair strategies using a variety of fibre-oriented repair geometries [41]. It was found that the different stepped joints recovered between 53% and 60% of their initial strength. These repair approaches are matured processes frequently used in industry. The here-tested repair approaches are novel with room for improvement. The repair mechanisms of the ADF vitrimer composite and the established thermoset differ strongly. Nonetheless, it highlights the potential of the vitrimer repair approaches for industrial applications.

During the tensile test, all specimens repaired with the full-patch approach broke in the same location as their virgin counterparts, as shown in Figure 5c,d. The failure mode was often a combination of fibre fracture and repair strip delamination. This indicated insufficient repair time which led to sub-optimal consolidation of the repair strip and the virgin material.

The local-patch repair approach on the other side broke in different locations to the initial failure as depicted in Figure 5a,b. The repaired region recovered mechanical properties comparable to the rest of the specimen, which indicates successful repair and adequate consolidation time.

One specimen of the local repair batch broke twice in the same region. This specimen showed a 30% increase in tensile strength compared to its virgin counterpart. This indicated successful repair of the fracture zone. It is important to note that the full-patch repair approach recovered the initial strength better than the local-patch repair approach. It is suspected that through fibre misalignment due to the longer consolidation time and the increased deformation, the local-patch repair approach reduced the mechanical properties of the specimen more than the reinforcement of the full-patch repair approach.

#### 4.2.2. Imaging Results

**Figure 7:**
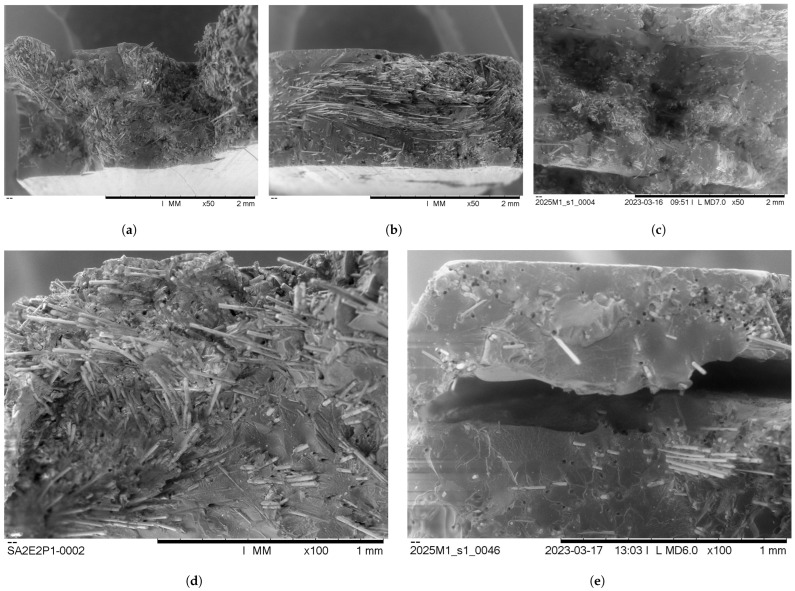
Scanning electron microscope (SEM) micrographs from various samples. (**a**) Virgin vitrimer–basalt specimen fracture zone x50. (**b**) Vitrimer–basalt local-patch repair fracture zone X50. (**c**) Vitrimer—basalt full-patch repair fracture zone X50. (**d**) Vitrimer–basalt local-patch repair fracture zone X100. (**e**) Vitrimer–basalt full-patch repair fracture zone X100.

The failure zones of the virgin and repaired specimens were analysed under the SEM; the images are shown in Figure 7. It was observed that in both repair cases, fibres were present in the failure area, which indicated successful repair and repeated interlocking of the ADFs.

For the full-patch repair, it is evident from Figure 7e that the number of fibres in the failure zone seemed to have reduced when compared to Figure 7a. Nonetheless, during the repair process, the ADFs interlocked again. This became clear by seeing fibres in the fracture zone and fibre pull-out events. As a result of the resin flow in the semi-closed mould, it seemed that the fibre volume fraction reduced as the material started to flow out. Furthermore, the delamination of the repair strips of the full-patch repair became clearly visible in Figure 7e.

Based on the SEM images shown in Figure 7a,b, the fibre volume fraction of the locally repaired specimens seems to be maintained, indicating favourable repair behaviour. However, it appears that in Figure 7d the ADFs seem to be at an out-of-plane angle compared to the virgin sample. It was suspected that, owing to the non-uniform consolidation pressure and geometrical deformation of the specimen, an out-of-plane misalignment was induced in the ADFs.

**Figure 8:**
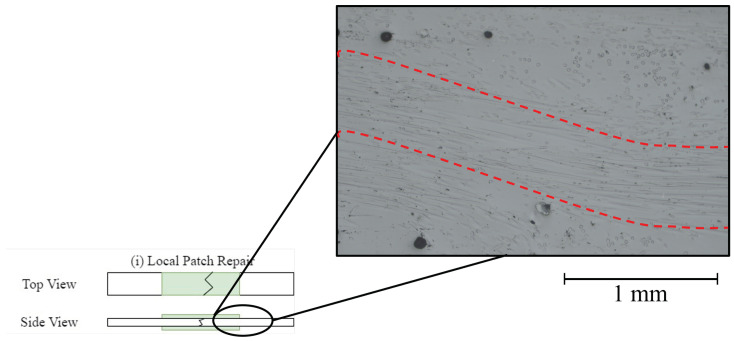
Micrograph of the polished side view of a local-patch repair specimen depicting the tendency of misalignment of the fibres highlighted by red dotted lines.

To further investigate how the fibre alignment was affected by the local-patch repair. Cross-section pieces at the edge of the repair strip were taken from various locally repaired specimens and polished as described in Section 4.1. The specimens showed a trend of waves in the through-thickness direction of the fibre alignment. An image of one of these waves is shown in Figure 8. This behaviour is typically observed for non-uniform consolidation pressure as described in the work performed by Belnoue et al. [42].

As the SEM images of the fractured cross-sections (Figure 7d) were analysed, it became clear that the fibres of the locally repaired specimens seemed to be aligned at an angle. This was further proven when the polished sections were investigated. These indicated that due to the resin flow and the non-uniform consolidation pressure of the local repair approach, waves of ADFs were formed. These can negatively affect the mechanical properties of the specimen, as the fibres are no longer aligned in the direction of the applied load, thus creating weaker regions, as shown in Figure 8, that can easily experience failure. Under the SEM, more fibre pull-out events were observed, which translates to reduced mechanical properties. The alignment of the fibres strongly depended on the amount of resin flow and the way the consolidation pressure was applied.

Based on the SEM images, it was concluded that the (i) repair approach compromised the ADFs’ alignments; approach (ii), on the other hand, maintained favourable fibre alignment at the cost of fibre volume fraction in the fracture area. The successful conservation of the fibre alignment was attributed to the reduced resin flow and uniformly applied consolidation pressure.

This was further underlined by the superior strength recovery of the full-patch repair specimens. It was shown that the local-patch approach had a larger amount of fibres in the fracture zone at the cost of fibre alignment. This was demonstrated by the lower strength of the normalised results and the SEM images. The full-patch approach, on the other hand, reduced the fibre volume fraction in the fracture area. However, the full-patch repair maintained a better alignment of the ADFs as seen by the high normalised strength recovery and the SEM images of Figure 7c.

It is important to note that the longer repair time and the initially increased deformation of the specimen played an important role in the local repair work: longer consolidation time allowed the repair strips and the virgin material to mix and nest together. This minimised the risk of delamination and preliminary failure. The increased flow of the resin was suspected to allow the ADFs to interconnect again and restore the initial strength. This came at the cost of reduced fibre alignment.

In contrast, Toyoda et al. investigated thermal welding repair of ultra-thin chopped carbon fibre tape-reinforced thermoplastics. During their research, it was observed that the tape reinforcements did not interlock again [43]. It was suspected that this is due to the longer nature of the fibres used and the ultrasonic welding. The advantage of vitrimer to thermoplastic patch repair is that vitrimeric resins require lower temperatures around 120 °C compared to the temperatures required for high-performance thermoplastic repair, such as poly ether ether ketone (PEEK), which is around 385 °C. This allows a way to achieve lower viscosity, better mouldability, and easier repair of the vitrimer material at a reduced cost. It is important to note that PEEK exhibits significantly superior mechanical properties than the vitrimeric resins in this study, which come hand in hand with a higher processing temperature. Currently, it is difficult to make a meaningful comparison of repair work using vitrimer to thermoplastic as there is only limited research on ADF repair using the latter. Furthermore, the repair mechanisms of the two material types differ; the vitrimer reforms cross-links, whereas the thermoplastic polymer chains rearrange themselves once heated.

Owing to the narrow nature of the specimens, the semi-closed mould imposed resin flow along the fibre direction. This helped the ADFs to maintain their alignment and to recover the mechanical properties of the composite. This highlights the superior repair potential of the ADFs compared to continuous fibres. The misalignment issue would become more apparent for wider specimens and fully open moulds, as resin flow is more likely to occur in the transverse fibre direction. This would lead to more significant fibre misalignment and reduction in the mechanical properties. The challenge this highlights has to be addressed to further ensure that this type of repair technology is ready for industry.

In comparison, Erland et al. performed repair tests on random discontinuous fibres with thermoplastic resin [44]. It was observed that the mechanical properties using several repair patches were fully recovered. It was acknowledged that the repair performance is strongly related to the absence of fibre alignment and that this could lead to further challenges.

The concept of using different repair approaches on ADFs was proven to work; the ADFs interlocked again, showing successful repair of the fracture zone. It was acknowledged that the repair of larger specimens could yield different repair mechanics that require further investigation. If the repair mould and the consolidation pressure can be tailored accordingly to the fibre orientation, there is great potential for future application of ADF repair using vitrimeric resins as well as thermoplastics.

It can be concluded that the local-patch repair will require more investigation to ensure better consolidation pressure and preservation of the fibre alignment. Conversely, the full-patch repair approach requires longer consolidation time to minimise the risk of delamination. The vitrimer resin is currently being further investigated to yield a more mature manufacturing approach. This will enable thinner resin film manufacturing and hence better laminar tailoring.

## 5. Conclusions

The micro-mechanical behaviour of two types of sizing for basalt fibres was successfully reported. This consisted of determining the fibre strength and the interfacial shear strength between the basalt fibre, an epoxy resin, and a novel vitrimeric resin. The vitrimeric resin was shown to match the performance of the epoxy resin.

It was proven that the basalt fibres are a suitable new material for the HiPerDiF manufacturing approach. When it came to the mechanical behaviour of the materials, the specimens were successfully manufactured and tested. The aligned discontinuous basalt fibres were successfully combined for the first time with the epoxy and vitrimer resin systems, separately. The normalised tensile test results indicated that the vitrimer–basalt specimens had comparable mechanical properties to their basalt epoxy counterparts. The fracture zones were analysed under the SEM and typical ADF failure mechanisms were observed.

Two repair approaches were tested for the ADFs vitrimer–basalt specimens: the local-patch and the full-patch repair. In both cases, the specimens were successfully repaired and recovered a significant amount of their initial strength. The fracture zones were investigated under an SEM. It was observed that the discontinuous fibres interlocked again and no dry patches were visible. Furthermore, it was concluded that the local repair methodology compromised the fibre alignment. Nonetheless, it recovered 34.0% and 56.1% of the initial strength for the type 1 and type 2 sizing. The full-patch repair approach had a reduced amount of fibres in the failure zone but maintained the high-quality of fibre alignment. It recovered 57.1% and 78.2% of the initial strength for the type 1 and type 2 sizing, respectively. Additionally, the full-patch repair better recovered the limit load than the local-patch repair approach. For future research, it would be advisable to use a longer consolidation time and to determine a method to ensure homogeneous consolidation pressure.

Finally, it is important to acknowledge that the T100 VITRIMAX resin is a relatively new material and its manufacturing process is not yet fully matured. With further research, a higher fibre volume fraction shall be more achievable. The differences between cured and melted vitrimer should be investigated through the examination of interfacial shear strength (IFSS) and tensile properties. Nonetheless, the high potential of the vitrimeric resin is clearly shown by the favourable mechanical properties yielded. Combining this with the flexibility in manufacturing and the successfully proven repair potential, the vitrimeric resin seems to be a sustainable competitor to standard epoxy matrices. This research underlines the potential of vitrimeric resins in composite applications to promote a circular economy, especially in lightweight applications such as in the logistical and automotive industries offering an alternative to traditional non-repairable epoxy matrices.

## Figures and Tables

**Table 1:** Single-fibre tensile test results, including the average fibre diameter, the failure strain, the elastic modulus, and the strength measured during the test. Errors ± represent the standard deviation from the mean.

Fibre Type	Gauge Length [mm]	Fibre Diameter [μm]	Failure Strain [%]	E-Modulus [GPa]	Strength [MPa]
Basalt Sizing Type 1	12±0.10	20.5±0.78	4.02±0.71	56.1±11.5	2003±380
Basalt Sizing Type 2	12±0.10	20.4±1.46	3.95±0.59	54.5±13.5	1987±362
HYBON 2026 Glass Comparison [35]	12±0.10	15	3.4	82	2790

**Table 2:** Microbond test results and critical fibre length—Mean interfacial shear strength ($\tau_{avg}$), interfacial shear strength values obtained from a linear fit ($\tau_{fit}$), the determined fibre strength, the critical fibre length ($l_{c}$), and the critical fibre aspect ratio ($AR_{c}$). Errors ± represent the standard deviation from the mean.

Fibre Resin Couple	$\tau_{\mathrm{avg}}$ [MPa]	$\tau_{\mathrm{fit}}$ [MPa]	Fibre Strength [MPa]	$l_{c}$ [mm]	${\mathrm{AR}}_{c}$ [mm/mm]
Sizing Type 1 Epoxy	37.7±7.66	36.9	2003±380	0.556	27.1
Sizing Type 2 Epoxy	36.6±5.74	37.1	1987±362	0.546	26.8
Sizing Type 1 Vitrimer	42.9±10.1	39.0	2003±380	0.526	25.7
Sizing Type 2 Vitrimer	51.2±5.53	51.0	1987±362	0.397	19.5

**Table 3:** Material aerial weight overview (g/m^2^) and the respective material density (g/cm^3^). Errors ± indicate the standard deviation from the mean.

Material Type	Aerial Weight [$g/m^{2}$]	Material Density [$g/{\mathrm{cm}}^{3}$]
Basalt Sizing Type 1 HiPerDiF	124±45.8	2.67
Basalt Sizing Type 2 HiPerDiF	125±17.7	2.67
VITRIMAX T100	200	1.05
K51 Epoxy Resin	43	1.21

**Table 4:** Normalised tensile test results and comparison of the virgin and repaired vitrimer–basalt specimens (stress–strain curves are given in Figures S3 and S4) listing the elastic modulus, the maximum strength, and the recovered strength as a percentage of the initial strength. Errors ± indicate the standard deviation from the mean.

Material Couple	Repair Method	E-Modulus V_f_ 38% [GPa]	Max Strength V_f_ 38% [MPa]	Recovered Strength [%]
Vitrimer Basalt Sizing Type 1	Virgin Specimen	29.7±2.91	396±32.3	Na
	Local-Patch Repair	20.4±1.57	149±30.3	34.0±11.2
	Full-Patch Repair	27.9±1.26	218±14.5	57.1±1.76
Vitrimer Basalt Sizing Type 2	Virgin Specimen	32.0±2.25	399±36.1	Na
	Local-Patch Repair	23.5±1.73	226±51.0	56.1±16.7
	Full-Patch Repair	35.6±2.22	326±39.6	78.2±9.14

## Data Availability

All underlying data are provided in this published article (and Supplementary Materials).

## Supplementary Materials

The following supporting information can be downloaded at: https://www.mdpi.com/article/10.3390/polym16081089/s1.

## Abbreviations

The following abbreviations are used in this manuscript:

ADF	Aligned Discontinuous Fibre
ADFRC	Aligned Discontinuous Fibre-Reinforced Composites
ADFRP	Aligned Discontinuous Fibre-Reinforced Polymer
ASTM	American Society for Testing and Materials
CAN	Covalent Adaptable Network
GSM	Grams per Square Metre
HiPerDiF	High-Performance Discontinuous Fibre
IFSS	Interfacial Shear Strength
MDPI	Multidisciplinary Digital Publishing Institute
DOAJ	Directory of Open Access journals
PEEK	Poly Ether Ether Ketone
SFTT	Single-Fibre Tensile Test
TGA	Thermo-Gravimetric Analysis
SEM	Scanning Electron Microscope
