# Supplementary material for: Mechanical Behaviour of As-Manufactured and Repaired Aligned Discontinuous Basalt Fibre-Reinforced Vitrimer Composites
Source: Polymers (Basel). 2024 Apr 13;16(8):1089. doi: 10.3390/polym16081089 (PMC11053685; doi:10.3390/polym16081089)
Supplement: Supplementary file 1 [file polymers-16-01089-s001.zip › polymers-2942553-supplementary.pdf]

Supporting information to

Mechanical Behaviour of As-Manufactured and Repaired Aligned Discontinuous Basalt Fibre Reinforced Vitrimer Composites

Leon Messmer, Ali Kandemir*, Burak Ogun Yavuz, Marco L. Longana, and Ian Hamerton

Bristol Composites Institute, School of Civil, Aerospace, and Design Engineering, University of Bristol, Queen’s Building, University Walk, Bristol, BS8 1TR, UK

*e-mail address: ali.kandemir@bristol.ac.uk

Polishing of the Virgin Specimens

The following figure S1 show the polishing result of the virgin material cross section. From the polished cross-section of the virgin vitrimer basalt specimen it can be clearly seen that initially the fibres are well aligned. This is seen as all the circles are close to perfectly circular as opposed to oval shapes that would indicate fibre misalignment. Furthermore, minimal dry spots are visible, and the fibres seem to be distributed in a homogeneous way through the cross section. This indicates that the ADF composite material was successfully manufactured.

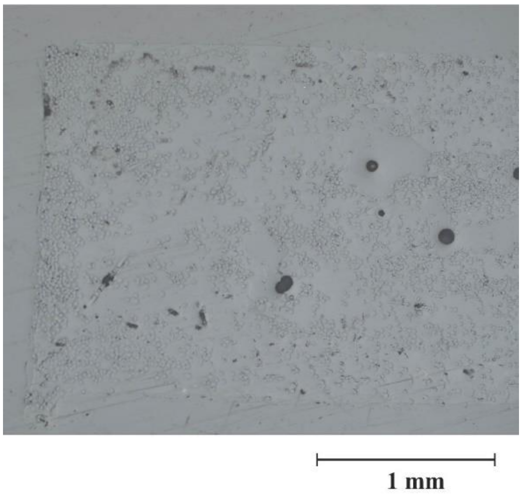

(a) – Virgin basalt type 1 sizing vitrimer specimen

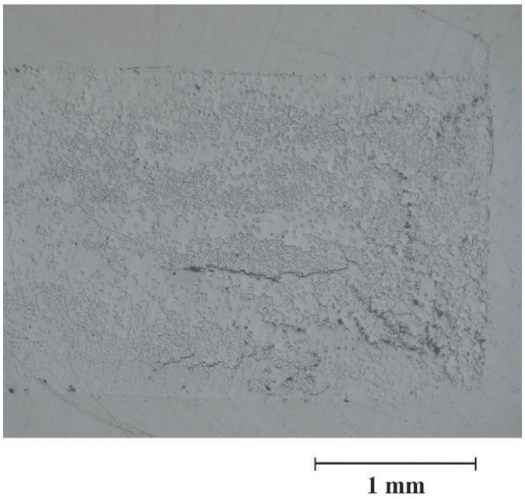

(b) – Virgin basalt type 2 sizing vitrimer specimen

Figure S1 – Micrograph depicting polished cross sections of virgin specimen.

Tensile test maximum load

The following table S1 illustrates the failure load of the various fibre matrix specimens and the failure strain.

Table S1 - The failure strain (%), the failure load (N), and the recovered maximum load for the various specimen types. The ± indicates the standard deviation from the mean.

Repair Approach	Material Couple	Failure Strain [%]	Failure Load [N]	Recovered Max Load [%]
Vitrimer Basalt Sizing Type 1	Virgin Specimen	1.45 ± 0.133	1730 ± 531	Na
	Local Patch Repaired	0.754 ± 0.172	666 ± 162	58.7 ± 19.2
	Full Patch Repaired	0.852 ± 0.074	1610 ± 95.6	74.6 ± 7.6
Vitrimer Basalt Sizing Type 2	Virgin Specimen	1.38 ± 0.181	1920 ± 229	Na
	Local Patch Repaired	1.12 ± 0.135	1490 ± 247	88.1 ± 25.9
	Full Patch Repaired	0.995 ± 0.068	1930 ± 185	94.5 ± 16.6

It became clear that even though the strength values of the repaired specimen reduced significantly, the limit load was mostly recovered. This was particularly pronounced when considering the full patch repair approach, recovering 74.6% and 94.5% of the limit load for the type 1 and 2 sizing respectively. This was associated with the fact that the entire specimen was reinforced. The local repair of the type 1 and 2 sizing also yielded a 58.7% and 88.1% recovery of its initial limit load. It is important to note that for all the locally repaired specimens that broke outside of the repaired area, the limit load was reduced. That reduction was traced back to the elongating of the specimen and the reduction in the alignment of the ADF due to the resin flow.

Microbond test results

The following figure S2 show the results of the microbond test of the various fibre matrix couples, the green circles, red square, and blue triangle represent, the successful debonding, droplet matrix failure, and fibre failure respectively. The successful debonding results were plotted and a linear fit having an intercept at 0 was applied to determine the interfacial facial shears stress (IFSS) (τ_{fit}). This was compared with the average IFSS of the droplets τ_{AvG}. Additionally, the 95% confidence interval was plotted.

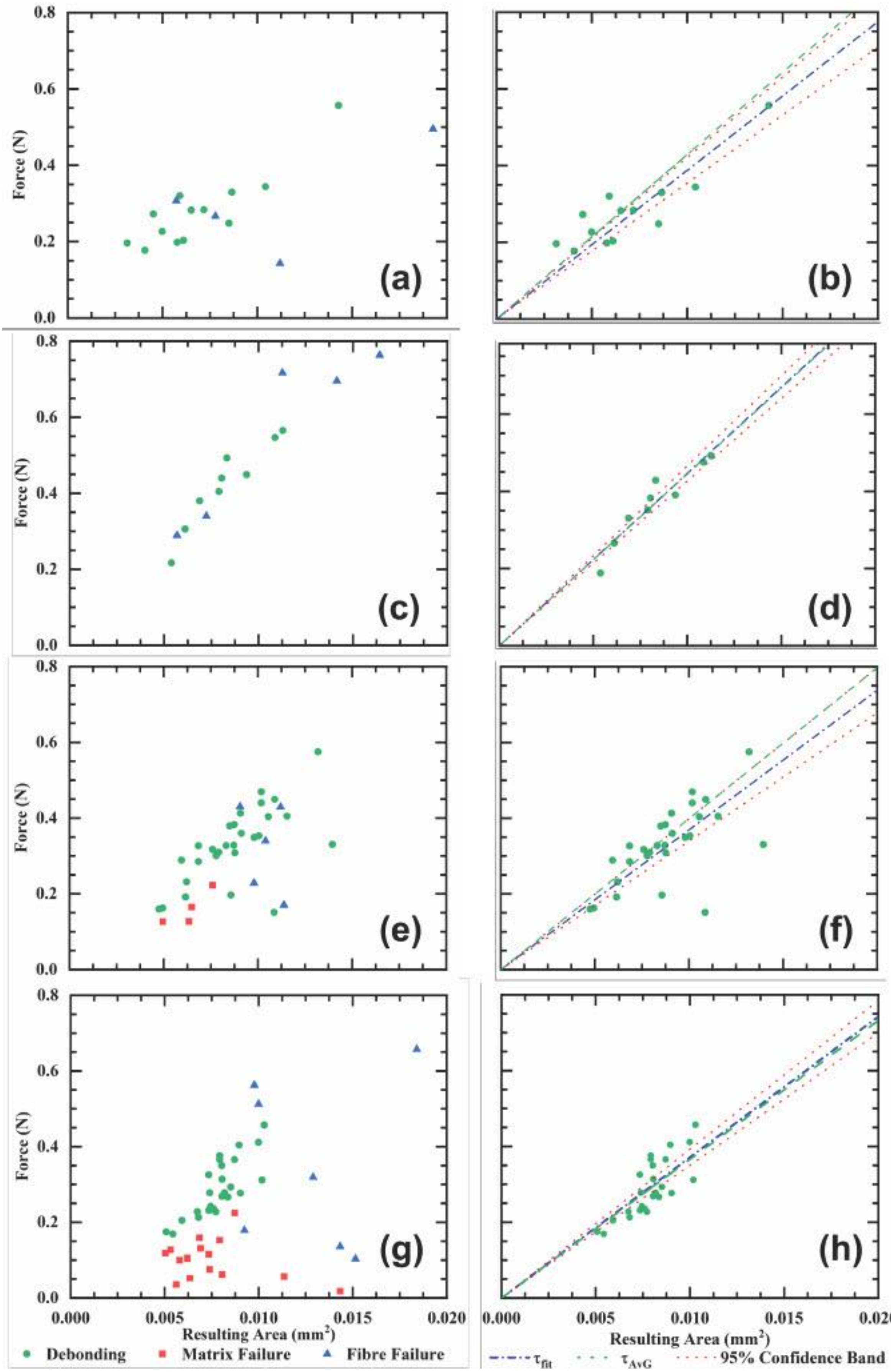

Figure S2 - Microbond test results - (a) and (b) vitrimer basalt sizing type 1 - (c) and (d) vitrimer basalt sizing type 2 - (e) and (f) epoxy basalt sizing type 1 - (g) and (h) epoxy basalt sizing type 2

The stress strain curves of the tensile test

The following figures (S3 and S4) show the stress strain curves for the various fibre matrix composites. Figure (S4) additionally shows the tensile test stress strain curves of the diverse repair procedures of the vitrimer composite material.

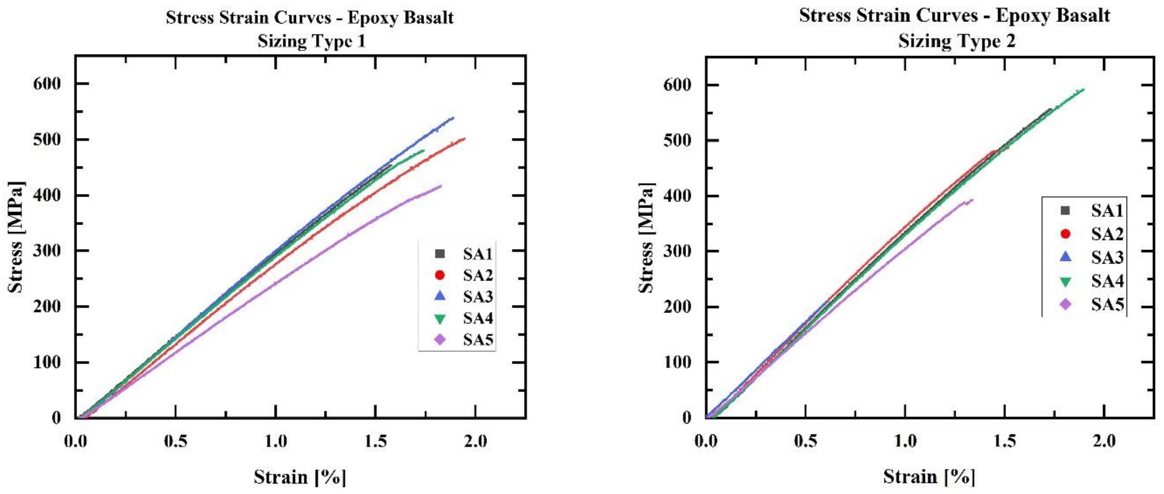

(a) - Epoxy basalt sizing type 1 **(b) -** Epoxy basalt sizing type 2

Figure S3 - Stress strain curves for the epoxy basalt specimens

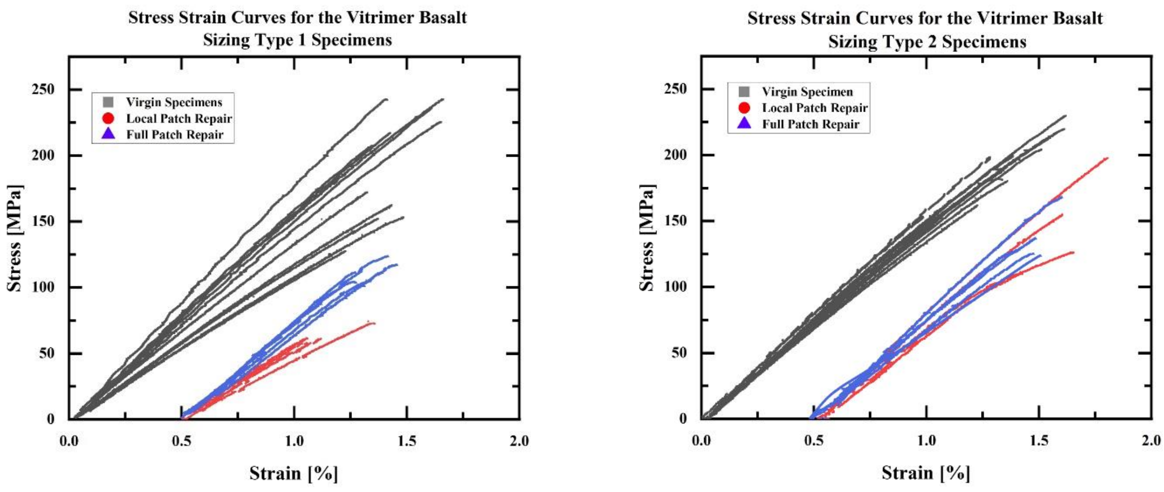

(a) - Vitrimer basalt sizing type 1 **(b) -** Vitrimer basalt sizing type 2

Figure S4 - Stress strain curves for the vitrimer basalt specimens – Comparing the virgin and repaired samples

TGA Results of the various composite specimens

The following table S2 represents the TGA results for the various fibre matrix couples and the different repair methodologies if applicable.

Table S2 - TGA Results showing the fibre volume fraction as a % for the various specimens categories. The ± sign indicates the standard deviation from the mean.

Material Type	TGA Fibre Volume Fraction [%]		
	Virgin	Local Repair	Full Repair
**Epoxy A76.9.2**	45.3 ± 3.00	Na	Na
**Epoxy A76.9.4**	53.0 ± 1.61	Na	Na
**Vitrimer A76.9.2**	17.8 ± 5.79	18.8 ± 3.57	19.5 ± 4.07
**Vitrimer A76.9.4**	18.2 ± 4.38	24.7 ± 2.53	16.3 ± 3.15

Based on the rule of mixture and the aerial density of the materials, the epoxy specimens resulted in an estimated fibre volume fraction of 36.2% and 36.4% for the sizing type 1 and 2 basalt respectively. The resulting fibre volume fraction for the vitrimer specimens was calculated to be around 16.9% and 17.0% for the sizing type 1 and 2 respectively. In contrast to the epoxy specimens, the fibre volume fraction of the vitrimer specimens is much lower due to the thicker nature of the vitrimer film. Furthermore, it was not desirable to stack several fibre preforms without having a resin strip in between as this could lead to misalignment, nesting, and poor wetting of the fibres. This limited the fibre volume fraction tailoring of the vitrimer manufacturing.

When comparing the predicted values to the TGA results from Table S2 it becomes clear that the epoxy specimens' fibre volume fraction was under-predicted by around 35%. The reason for that is that due to the low viscosity of the epoxy resin, a significant amount has bled out the mould. In contrast the fibre volume fraction of the vitrimer basalt was only under predicted by around 6% due to the higher viscosity of the vitrimeric resin.
